# Preparation and Characterization of Soybean Oil-Based Polyurethanes for Digital Doming Applications

**DOI:** 10.3390/ma10080848

**Published:** 2017-07-25

**Authors:** Vincenzo Pantone, Amelita Grazia Laurenza, Cosimo Annese, Roberto Comparelli, Francesco Fracassi, Paola Fini, Angelo Nacci, Antonella Russo, Caterina Fusco, Lucia D’Accolti

**Affiliations:** 1 Greenswitch s.r.l., 71013 Ferrandina (MT), Italy; vincenzo.pantone@greenswitch.it (V.P.); antonella.russo@greenswitch.it (A.R.); 2 Dipartimento di Chimica, Università di Bari “A. Moro”, Via Orabona 4, 70126 Bari, Italy; amelitalaurenza@gmail.com (A.G.L.); francesco.fracassi@uniba.it (F.F.); angelo.nacci@uniba.it (A.N.); 3 ICCOM-CNR, SS Bari, Via Orabona 4, 70126 Bari, Italy; annese@ba.iccom.cnr.it; 4 IPCF-CNR, SS Bari, Via E. Orabona 4, 70125 Bari, Italy; r.comparelli@ba.ipcf.cnr.it (R.C.); p.fini@ba.ipcf.cnr.it (P.F.)

**Keywords:** catalysis, soybean oil, bio-based polyurethane, doming application

## Abstract

Polyurethane-resin doming is currently one of the fastest growing markets in the field of industrial graphics and product identification. Semi-rigid bio-based polyurethanes were prepared deriving from soybean oil as a valuable alternative to fossil materials for digital doming and applied to digital mosaic technology. Bio-resins produced can favorably compete with the analogous fossil polymers, giving high-quality surface coatings (ascertained by SEM analyses). In addition, polyurethane synthesis was accomplished by using a mercury- and tin-free catalyst (the commercially available zinc derivative K22) bringing significant benefits in terms of cost efficiency and eco-sustainability.

## 1. Introduction

Since 1937, pioneering works regarding to the polyurethanes (PU) [1] show that these polymers can be obtained with different properties ranging from rigid glasses to flexible foams, and used as elastomers, adhesives, coatings, resins, and so on [2,3,4,5]. Polyurethane linkages are usually obtained through the reaction of polyols with isocyanates, using hazardous Lewis acids as catalysts (e.g., mercury-compounds) and/or toxic bases (e.g., tertiary amines) [6,7,8]. Over recent years, the Hg compounds have suffered from severe restrictions by the European Chemical Agency (ECHA), while the derivatives of Sn (IV) are still admitted although equally toxic [9].

A special application of PU is digital doming, a process which adds value to any shape or size of non-porous material by coating the surface with a thick layer of resin (up to 5 mm) having a dome shape, which is due to the high surface tension of the liquid polymer during solidification. This technique, which gives to the surface a three-dimensional effect, is used for a variety of applications, such as key chains, stickers and nameplates. Polyurethane-resin doming is currently one of the fastest growing markets in the field of industrial graphics and product identification, as PU resins are very durable, tough long-lasting polymers that cannot be easily scratched or dented. In addition, a good-quality resin does not yellow when exposed to UV and presents no health and safety issues both in production and final application of the cured domed object [10].

**Figure 1:**
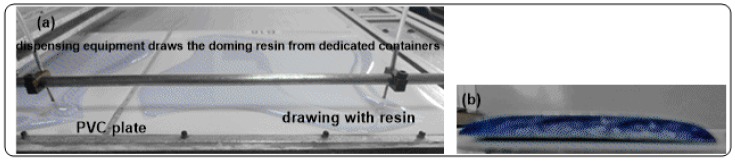
Example of Mosaico Digitale^®^ (Pepe & CO, Gravina, BA, Italy)) tile prepared with polyurethane (**PU_MD**) deriving from fossil Domes polyol: (**a**) Deposition of Tile on PVC; (**b**) section of piece of tile: The “mosaico digitale” technology uses three components: (i) the resin, from which the dome or lens is formed, (ii) the dispensing equipment, that receives the resin from dedicated containers and draws it on the support surface and (iii) a support, in this case a PVC foil, that receives the resin during the “pour time”.

An example of digital doming is the preparation of PVC tiles covered with polyurethane coating useful for the decoration of walls (Figure 1).

Most of the worldwide market plastics derive from fossil fuels such as oil, coal and natural gas, accounting for about 7% of world consumption of these resources. Considering the continuous depletion of fossil raw materials, the oil price fluctuations and environmental problems (e.g., non-recyclable toxic wastes, CO_2_ emissions and climate change), in the last decade a wide-ranging research started on the production of “bio-based” polymeric materials coming directly or indirectly from renewable raw materials such as starch, cellulose, sugars, lignin, etc. [11,12].

In this context, vegetable oils, such as castor, linseed, and soybean oils, have been regarded as a convenient renewable feedstock for developing bio-plastics [13,14,15,16,17].

**Figure 2:**
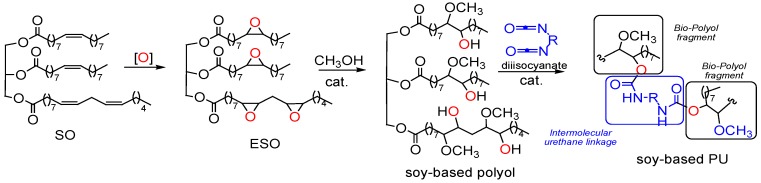
Scheme of general synthesis of soy-based PU.

Our twenty years’ experience in green chemistry [18,19,20,21,22,23] has convinced us to extend our studies to the synthesis of bio-polyurethanes [24]. In fact, in our recent work we just reported an efficient, cost-effective, and environmentally safer conversion of soybean oil (SO) into soy-based PU, using as catalyst the molybdenum(VI) dichloride dioxide (MoCl_2_O_2_) for one-pot synthesis starting from the epoxidized oil (ESO) and with 2,6-tolyl-diisocyanate (TDI). (Figure 2).

Considering the results obtained, we decided to extend our findings to the digital doming technique, for which, to the best of our knowledge, only a few recent studies relate to the chemical-physical properties of these resins petroleum-based [25,26,27,28], while no examples on the use of bio-based PU resins have been reported until now.

Investigations were carried out addressing two main issues: (i) the search for suitable eco-friendly polymerization catalysts, based mainly on Zn(II) and Sn (IV) derivatives, for producing mercury-free polyurethanes; and (ii) finding new soy-based PU resin formulations for preparing digital doming coatings of PVC tiles alternative to the petrochemical ones.

All new materials were characterized with DSC and SEM techniques to assess their chemical and morphological properties. The surface hydrophilicity or hydrophobicity has been determined using the contact angle [29,30].

## 2. Results and Discussion

Investigations started with the preparation and characterization of a petrochemical polyurethane coming from the commercially available model polyol Domes Resin HGFP40702 (20 g, containing the catalyst) and Isophorone diisocyanate prepolymer (IPDI prep.) (20.5 g), according to a known formulation already used industrially for the preparation of decorating mosaics (Figure 1).

The thus obtained polyurethane (labeled as **PU_MD**) started to cure after 60 min and finished in 12 h. The wetting contact angle (WCA), a crucial parameter for doming application being correlated to the surface tension, was evaluated to be 77° indicating a hydrophilic surface [29]. DSC analyses of **PU_MD** showed a glass transition temperatures *Tg* = 59 °C, suggesting that polyurethane sample is a glass at room temperature. The density value was found to be 1.01 kg/dm^3^.

**Figure 3:**
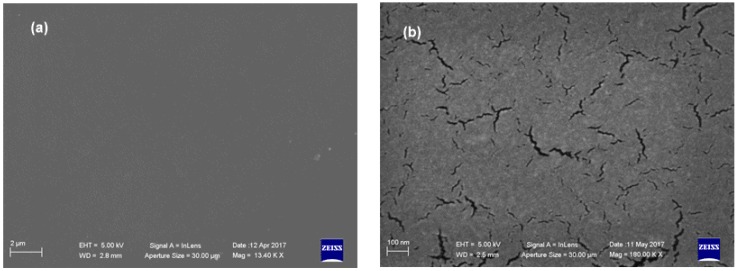
SEM microphotographs of **PU_MD** at different magnification: (**a**) 13 Kx and (**b**) 180 Kx.

Surface morphology, ascertained by SEM analyses, showed that the **PU_MD** possesses a homogeneous surface that allows the uniform covering of the piece of the tile (Figure 3a). Some cracks can be observed in higher magnification image (Figure 3b).

**Figure 4:**
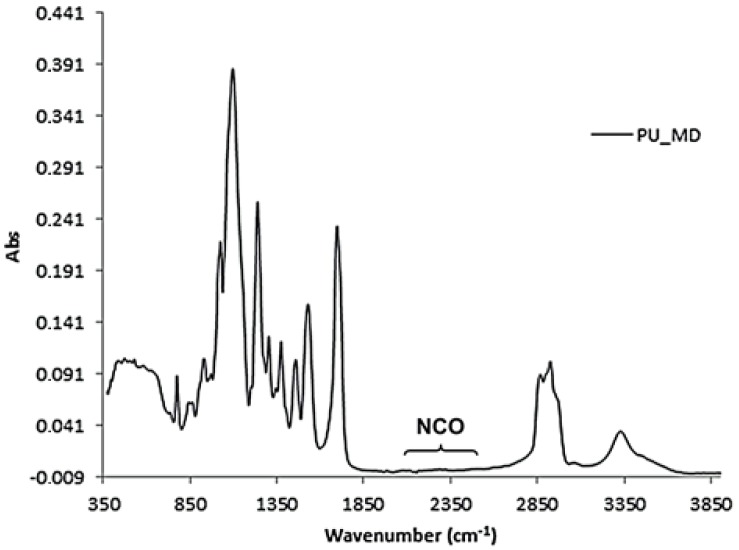
IR spectrum of **PU_MD**.

Analysis of IR spectra of **PU_MD** after 12 h revealed the presence of functional groups characteristic of polyurethane, in particular NH stretching band at 3400–3350 cm^−1^ and C=O stretching band at 1683 cm^−1^. The absence of the stretching band of isocyanate group at 2300–2250 cm^−1^ ensures the complete polymerization. (Figure 4).

With the aim of preparing the analogous bio-based PU possessing physic-chemical and morphological properties like those of **PU_MD**, we attempted to replace the fossil polyol Domes Resin HGFP40702 with the bio-polyol coming from methanolysis of ESO. This choice was based on the similarity of the physical and chemical properties of the two compounds, specifically on the values of viscosity, density and the hydroxyl group number (~3.5 -OH groups per molecule).

In first instance, the fossil polyol was completely replaced with its bio-based counterpart, the formulations were carried out using 20 g of **Bio-Polyol**, 10.3 g of IPDI prepolymer (OH:NCO 1:1.1 molar ratio) and variable loadings (from 0.2% to 0.5% *w*/*w*) of DABCO T-12 (dibutyltin dilaurate DBTDL), a catalyst which is known to efficiently promote the urethane bond forming reactions [6,8].

Under these conditions, polymerization time at room temperature was the same as that of **PU_MD** (12 h), but the Bio-PU product (labeled as **BioPU_100**) proved to be rubbery at room temperature, with *Tg* = 9 °C, WCA = 71° and the density value of 1.22 kg/dm^3^.

**Figure 5:**
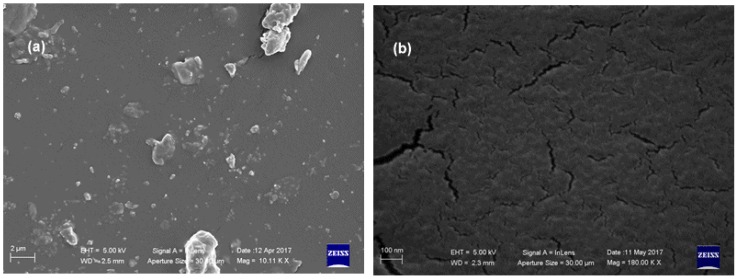
SEM microphotographs of **BioPU_100** at different magnifications: (**a**) 10 Kx; (**b**) 180 Kx.

SEM micrographs showed a significant change in the surface morphology, evidenced by the formation of agglomerates, which impede formation of a homogeneous surface (Figure 5).

To solve the problems of low *Tg* value (rubbery properties) and the poor surface homogeneity of **BioPU_100**, most probably due to the flexible alkyl chains of bio-polyol fragment, we decided to re-formulate by adding to the bio-based polyol a petrochemical fraction that would produce a PU with more similar properties to **PU_MD**. Indeed, according to international standards statements a polymer can be labeled as “bio-based” if its “bio-carbon percentage” is at least 20–50% (see experimental section) [31].

### 2.1. Selection of Fossil Polyol

With the aim of selecting the appropriate fossil polyol to be mixed with the analogous bio-based one, we carried out a screening among several homo-dispersed commercial polyols (Alcupol) displaying physic-chemical properties similar to Domes Resin HGFP40702 (experimental section). Selection of polyol was performed based on the properties of polyurethanes obtained by formulation with IPDI prepolymer, using DABCO-T12 as catalyst (Table 1).

Results of the screening clearly evidenced that all polyurethanes produced were hydrophilic, as demonstrated by WCA values falling in the range 71°–83°. As expected, *Tg* values were strictly dependent on the hydroxyl number of the starting polyols, with the highest values for Alcupol C5710 (50 °C) and Alcupol R3810 (45 °C), that are also the most similar values to those of **PU_MD** (Table 1, runs 2–3).

For this reason, and due to the similarity of density values, the two reagents Alcupol C5710 and R3810 immediately appeared as the most suitable fossil polyol counterpart for preparing Bio-PU for doming applications. The final choice was based on the WCA value of the two polyurethane products **PU_5710/T12** (WCA 71°) and **PU_R3810/T12** (WCA 79°) much closer to that of model **PU_MD** (WCA 77°) in the case of the latter polymer (Table 1, runs 1–3). Accordingly, Alcupol R3810 was the fossil polyol used for the successive formulations.

### 2.2. Catalyst and Loading Selection

Petrochemical polyol Alcupol R3810 was chosen also for selecting the suitable catalyst for the synthesis of our PUs. It is well known that organo-mercuric compounds are generally used as catalysts in polyurethanes production, mainly in non-foaming applications where little or no blowing reaction are required [32]. However, toxicity of mercury derivatives and other heavy metals has led to the development of alternative catalysts.

For our purposes, we chose organometallic compounds of Sn and Zn, less toxic than Hg and widely used as catalysts in the polyurethane industry. Selection was based on the evaluation of the cure time of polymerization reaction between Alcupol R3810 and IPDI prepolymer, assuming that this time should not exceed the twelve hours required for the complete polymerization of **PU_MD**.

**Figure 6:**
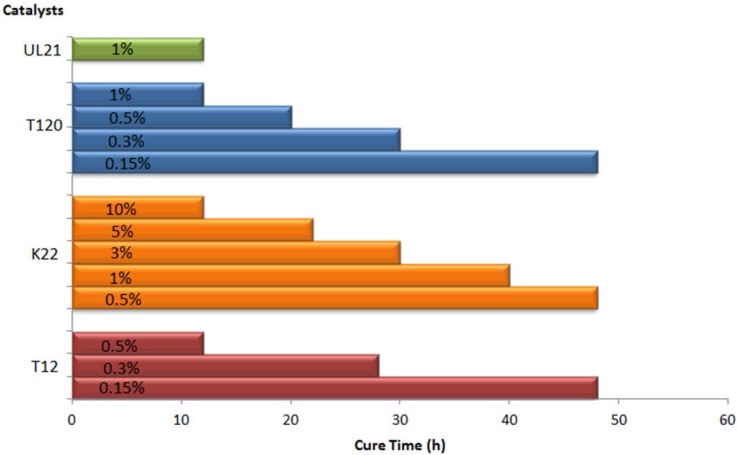
Cure times of PU production for some commercial Sn and Zn catalysts. Formulation conditions: 20 g of Alcupol R3810, 22.2 g of IPDI prepolymer, temperature 25 °C. Catalysts: T12 = dibutyltin dilaurate (DBTDL) [DABCO T-12]; K22 = Zinc Bis(2-ethylhexanoate) [Borchi^®^ Kat 22]; T120 = Dibutyl-bis(dodecylthio) stannane [T120]; UL21 = *O*,*O*′-bis(2-ethylhexyl)-(nonyl(octyl)stannanediyl) bis(carbonothioate) [UL21].

Investigations were also extended to the catalyst loading, which was evaluated by means of a set of experiments carried out by varying the amount of almost all the catalysts in the range 0.15–10% *w*/*w* respect to polyol (Figure 6).

From results in Figure 6 emerged that tin based compounds are the most active catalysts, with DABCO-T12 as the most efficient one. However, although the zinc derivative Borchi^®^ Kat 22 displays an activity 20 times lower than DABCO-T12, the former can be suggested as more suitable catalyst due to its much lower toxicity according to the ECHA [9].

### 2.3. Selection of Diisocyanate

The preliminary screening aimed at selecting the best set of reactants (polyols and catalyst) was extended also to search for the best diisocyanate. IPDI prepolymers are widely used for the synthesis of rigid polyurethanes, due to the presence of cyclohexane ring, but they can also be replaced with more flexible linear aliphatic diisocyanates such as 1,6-Hexamethylene diisocyanate (HMDI) and Dicyclohexylmethane-4,4′-diisocyanate (H12MDI) (Table 2) [29,33].

We tested both these reactants together with pure IPDI, evaluating the influence of their skeleton on the properties of the tile, with special attention to *Tg* and WCA values.

From data in Table 2 clearly emerged that all polyurethanes produced were hydrophilic in nature, as demonstrated by WCA values falling in the range 72°–79°. In addition, the highest *Tg* values were observed for PUs coming from diisocyanate H12MDI and pure IPDI (Table 3, runs 1–2), while the one deriving from HMDI was a rubber at room temperature (Table 2, run 3).

**Figure 7:**
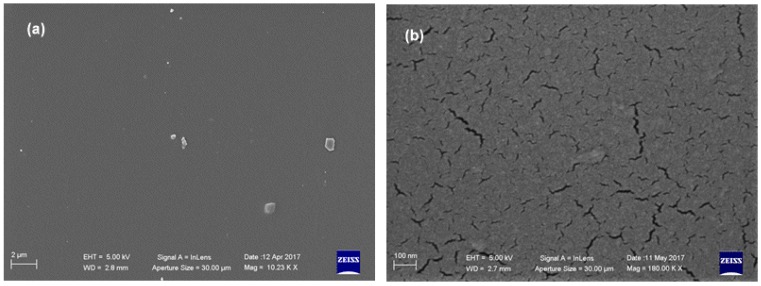
SEM microphotographs of **PU_3810/K22/H12MDI** at different magnifications: (**a**) 10 Kx; (**b**) 180 Kx.

The polyurethane coming from Dicyclohexylmethane-4,4′-diisocyanate (H12MDI) was chosen for the subsequent formulations, due to its physical proprieties in accordance with those of the model polyurethane **PU_MD** and because this diisocyanate has been recently used successfully for coating application [29]. SEM analyses pointed out the good coating quality of the produced polyurethane (**PU_3810/K22/H12MDI**) evidenced by the homogeneous surface (Figure 7).

### 2.4. Formulations of Bio-Based Polyurethane

With the whole set of selected reactants in hand (soy-based polyol, fossil Alcupol R3810, diisocyanate H12MDI, and catalysts K22), we searched for the best formulation of Bio-based polyurethane for doming applications, by mixing in several ratios the soy-based Bio-polyol and its fossil counterpart (Alcupol R3810) [33,34].

Based on International standards statements for labeling a product as “bio-based” (it must be composed of at least 20–50% of carbon atoms from renewable sources) [31], we produced a series of polyurethanes (namely **BIOPU_%C**) possessing the bio-component percentage in the range of 11–31% (Table 3).

From data reported in Table 3 emerged that all bio-polyurethane products possess physical properties (*Tg*, WCA and density values) much closer to those of the model (totally fossil) **PU_MD**.

**Figure 8:**
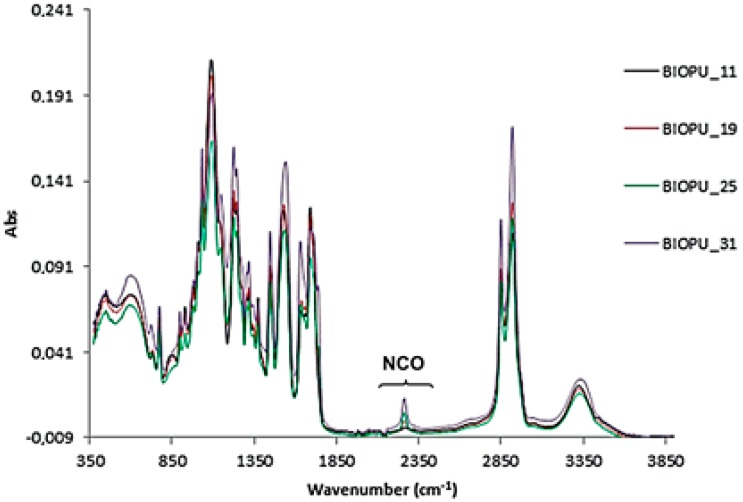
IR analysis of Bio-polyurethanes after 12 h.

The curing time was also checked by IR spectroscopy. The very low intensity of the stretching band at 2274 cm^−1^, due to isocyanate group, indicates that for the several Bio-PU investigated the curing process was almost complete in the expected time of 12 h (Figure 8).

**Figure 9:**
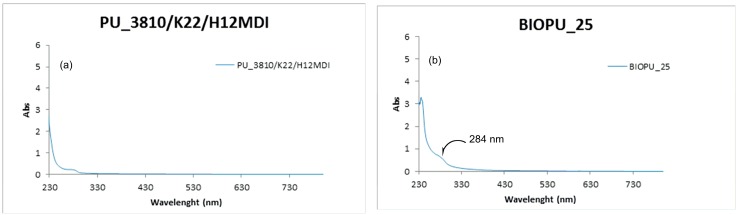
UV spectra for PU_3810/K22/H12MDI (**a**) and BIOPU_25 (**b**).

To verify that no change in transparency or clarity of PU has occurred after addition of the soy-based polyol we compared the UV-visible spectra of polyurethanes **BIOPU_25** and PU_3810/K22/H12MDI in the range of 200–800 nm. As shown in Figure 9, both polymers display very similar absorption spectra with a weak signal in the near UV (centered at 289 nm) and no absorption in the visible region (Figure 9).

**Figure 10:**
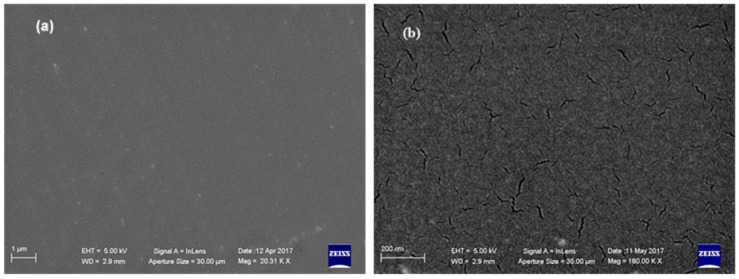
SEM microphotographs of **BIOPU_25** at different magnifications: (**a**) 20 Kx; (**b**) 180 Kx.

Interestingly, SEM analyses revealed that **BIOPU_25**, a bio-polyurethane that can be surely labeled as “bio-based”, displays a superior quality of surface morphology respect to **PU_MD**. In fact, besides the homogeneous coating that allows uniform covering of the piece of the tile (Figure 10a), it also displays, in contrast to **PU_MD** (Figure 3b), the absence of cracks as can be observed in the higher magnification image of Figure 10b.

This different morphology can be due to the foaming process caused by atmosphere moisture at the bond surface of polyurethane. This phenomenon, known also as “surface crazing”, produces deep cracks and splits through the polymer, with the depth of cracking increasing with greater extension [35].

This occurrence can be directly connected with the hydrophilic properties of the reacting mixture, and particularly with those of the starting polyol. Therefore, it is expected that the more hydrophilic fossil polyether polyol composing **PU_MD** gives rise to surface crazing in a greater extent respect to the bio-polyol of **BIOPU_25** bearing the more lipophilic aliphatic long chains of soybean oil.

## 3. Experimental

### 3.1. Materials and Methods

Polymerization catalysts dibutyltin dilaurate (DBTDL, DABCO T-12), dibutyl-bis(dodecylthio) stannane (T120), *O*,*O*′-bis(2-ethylhexyl)-(nonyl(octyl)stannanediyl) bis(carbonothioate) (UL21) and Zinc bis (2-ethylhexanoate) (Borchi^®^ Kat 22) were supplied from Borchers GmbH (Langenfeld, Germany), Evonik Degussa Italia (Pandino, CR, ITALT) and Momentive Performance Materials Inc (Waterford, NY, USA).

Petrochemical polyether polyols alcupol (Table 4) were supplied by S.p.A. (Rho, MI, Italy) (each of them as a homo-dispersed polyol possessing n. OH/molecule = 3). Domes resin HGFP40702 was a mixture of polyether polyols with different chain length containing also phenylmercury neodecanoate (0.3–0.8% *w*/*w*) having the following properties: OH/molecule = 3, n. OH (mgKOH/g) 398, density 1.04 (kg/dm^3^), viscosity 550 cP: IPDI prepolymer (a mixture of pure isophorone diisocyanate and domes resin). Both were a gift by Greenswitch Industry (Ferrandina, Matera, Italy).

1,6-hexamethylene diisocyanate (HDI), pure isophorone diisocyanate (IPDI), dicyclohexylmethane-4,4′-diisocyanate (H12MDI) were purchase from Evonik. Soybean oil (SO) was procured from the local market and used without any further purification (iodine number of 130 g I_2_/100 g). Epoxidation of Soybean oil (SBO) and Methanolysis of ESO using HBF_4_ were carried out following the literature procedure [24]. Chemical properties of ESO were reported: number of oxirane oxygen 6.3 g O/100 g; number of epoxy groups 4 for molecule; number of Iodine 1.5 g I_2_/100 g, number of double bonds/molecule 0.05 and Mn (g/mol) 943.807.

Chemical properties of Bio-polyol are the following: colorless liquid, Mn (g/mol) 1054.207: average number of bio-carbon 56.232 corresponding to 674.840 g/mol, average number of carbon deriving from methanol 3.45 corresponding to 41.437 g/mol of carbon, spectral data in agreement with literature [24]. Density (kg/dm^3^) 1.00176, viscosity (cP) 4550, n. OH (mg KOH/g) 191, n. OH/molecule 3.45. The NMR spectra were in agreement with literature [24].

### 3.2. Characterization Techniques

NMR spectra were recorded on an Agilent Technologies 500 MHz spectrometer (Agilent Company, Santa Clara, CA, USA); the ^1^H-NMR spectra (500 MHz) were referenced to residual isotopic impurity of CDCl_3_ (7.26 ppm).

A Viscometer Cannon-Fenske reverse flows YNT instrument (CANNON Instrument Company^®^, State College, PA, USA) was used to determine the viscosity using the standard method [36] 

The determination of density was carried out with the pycnometer method [37] Iodine number was determined with EN ISO 661 method, [38] oxirane number (ON) was determined with ASTM D 1652 method [39] and hydroxyl number values was determined with ASTM D4274 method [40] for all pre-polymers [24].

DSC analyses were performed on a Q200 TA Instruments apparatus (TA Instruments, New Castle, DE, USA). Experiments were carried out using the temperature range from −80 °C to 120 °C with a heating rate of 10 °C/min, under N_2_ gas at a flow rate of 30 mL/min [24].

The FE-SEM analyses were conducted with a Zeiss Sigma Field-emission scanning electron microscope instrument (Zeiss International, Oberkochen, Germany) operating at 5 kV and equipped with in-lens secondary electron detector. Samples were mounted onto stainless steel sample holders by double-sided carbon tape and gold sputtered prior to analysis.

Water Contact Angle (WCA) measurements were carried out by means of a CAM200 digital goniometer (KSV Instruments, Biolin Scientific SE-426 77, Västra Frölunda, Sweden) equipped with a BASLER A60f camera (KSV Instruments, Biolin Scientific SE-426 77, Västra Frölunda, Sweden). WCA values were determined using the sessile drop method with a double distilled water droplet of 1 µL (five measurements for each sample). In this case, the liquid is water, thus if a contact angle is greater than 90°, the solid surface is called “hydrophobic surface”, if the contact angle is less than 90° the surface is called “hydrophilic surface”.

Infrared absorption spectra of **PU_MD** and all **BIO_PU** were acquired in the range 375–4000 cm^−1^ with a resolution of 4 cm^−1^, using a vacuum Bruker Vertex 70v Fourier transform infrared (FTIR) spectrometer (Buker, Milan, Italy). Spectra were collected in attenuate total reflectance (ATR) mode utilizing an ATR module (Bruker Platinum ATR) equipped with a single reflection diamond ATR crystal (refractive index of 2.4).

UV-Vis absorption spectra were recorded with a UV-Vis-near IR Cary 5 Varian spectrophotometer (Agilent Technologies, Inc., Santa Clara, CA, USA) equipped with a coating support.

### 3.3. Digital Doming Polyurethane Synthesis

**Figure 11:**
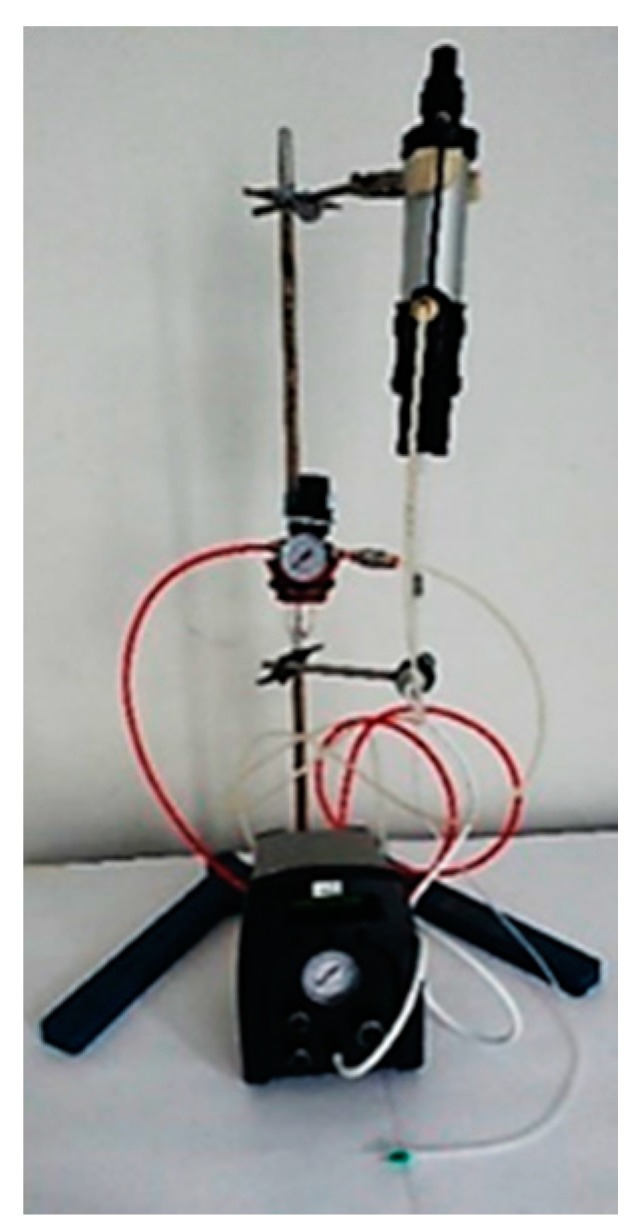
Volumetric precision low pressure doser.

Polyol (20 g) and catalyst (0.15–10% *w*/*w* respect to polyol) were mechanically stirred for 60 s to ensure their complete homogenization. After that, diisocyanate (or IPDI prepolymer) (OH: NCO 1:1.1 molar ratio) was added to the mixture and mechanically stirred for 2 min into a plastic container (pour time) [24]. Alternatively, reaction was performed by using a volumetric precision low-pressure doser consisting of two cylindrical reservoirs containing polyols and diisocyanate. After degassing, reactants are pumped into a static mixer and then deposited on the PVC layer through a nozzle (Figure 11). The gel times at room temperature for each compound were 4–12 min, while the polymerization time was 12 h.

### 3.4. Bio-PU Synthesis and Determination of Bio-Carbon Percentage

Procedure for the synthesis of Bio-PU was the same as that of the fossil resin, but in this case polyolic reactant was preliminarily prepared by mixing the proper amounts of fossil polyol (Alcupol) and soy-based Bio-polyol and stirring them until obtaining a homogeneous mixture.

A typical calculation for determining the bio-carbon percentage of Bio-PU, considering the several polymer components, is carried out according to Equation (1):

(1) $$\%C=\frac{n_{Bp}\;\times \;674.840\;g/\mathrm{mol}}{(n_{Bp}\;\times \;674.840\;\frac{g}{\mathrm{mol}})\;+\left(n_{met}\;\times \;41.437\frac{g}{\mathrm{mol}}\right)\;+(n_{Fp}\;\times \;144.128\frac{g}{\mathrm{mol}})\;+\;(n_{NCO}\;\times \;180.160\frac{g}{\mathrm{mol}})\;+\left(n_{cat}\;\times \;192.171\frac{g}{\mathrm{mol}}\right)}$$

where *n_Bp_* is the moles number of Bio-polyol component (soy-based polyol, Mn 1054.207 g/mol, average number of bio-carbon 56.232, corresponding to 674.840 g/mol), *n_met_* is the moles number of methanol component (based on hydroxyl number of 191 mgKOH/g, equivalent to 3.45 -OH/molecule and thus to 3.45 -OCH_3_/molecule, corresponding to 41.437 g/mol of carbon), *n_Fp_* is the moles number of fossil polyol component (Alcupol R3810, MF C_12_H_44_O_9_, MW 440.3 g/mol, corresponding to 144.128 g/mol of carbon), *n_NCO_* is the moles number of diisocyanate component (H12MDI dicyclohexylmethane-4,4′-diisocyanate, MF C_15_H_22_N_2_O_2_, MW 262.17 g/mol, corresponding to 180.160 g/mol of carbon) and n_cat_ is the moles number of catalyst component (K22, MF C_16_H_30_O_4_Zn, MW 350.14 g/mol, corresponding to 192.171 g/mol of carbon).

## 4. Conclusions

**Figure 12:**
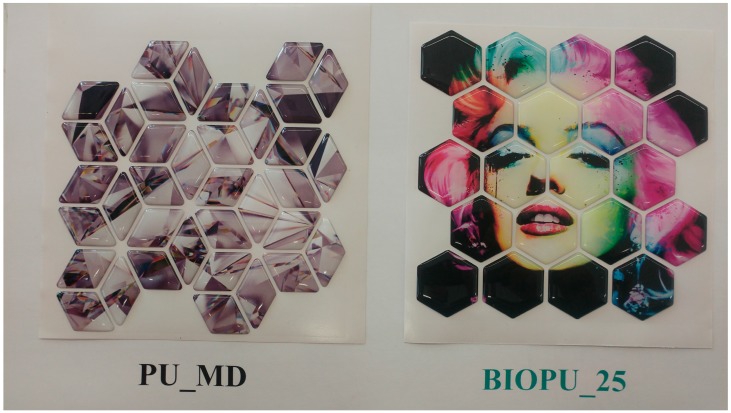
Comparison of tile obtained with domes resin (**PU_MD**) and **BIOPU_25** (picture kindly provided by Greenswitch Company, IT).

An innovative bio-based polyurethane deriving from soybean oil has been developed and for the first time applied to digital doming using mosaico digitale^®^ technology. Results of this work demonstrate that these bio-based polyurethanes can favorably compete with analogous fossil polymers in doming applications, and open the way to the substitution of petrochemical materials widely used in the field of decorations, such as those shown in Figure 12 depicting an example of tiles commercially using resin and the synthesized bio-based polyurethane.

Results of this work also allowed the two following advances: (i) the efficient synthesis of mercury- and tin-free bio-polyurethanes based on a clean and eco-friendly Zinc catalyst (K22) and (ii) the fully characterization of commercial digital doming tiles of fossil **PU_MD**, by measuring for the first time in the literature the physic-chemical properties (*Tg*, WCA and density) and surface morphology. All these improvements indicate that this new formulation of soy-based polyurethanes, which has no precedent in the literature, brings significant benefits in terms of cost efficiency and eco-sustainability and has a good potential for industrial applications.

## Figures and Tables

**Table 1:** Screening of commercial polyols ^a^.

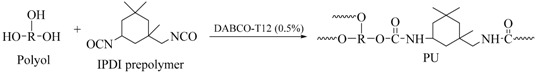								
Run	Polyol	M_n_ (g/mol)	n. OH (mgKOH/g)	IPDI Prep (g)	PU Products	*Tg* ^b^ (°C)	WCA ^c^ (°)	d (kg/dm^3^)
1	Domes Resin	-	398	20.5	**PU_MD**	59	77	1.01
2	Alcupol C5710	290	565	33.6	**PU_C5710/T12**	50	71	1.06
3	Alcupol R3810	440	380	22.2	**PU_R3810/T12**	45	79	1.06
4	Alcupol R2510	670	240	14.6	**PU_R2510/T12**	2	78	1.12
5	Alcupol R1610	1050	155	9.3	**PU_R1610/T12**	−27	79	1.49
6	Alcupol C3531	4800	35	2.1	**PU_C3531/T12**	−62	83	1.38

^a^ Formulation conditions: Polyol (20 g) and catalyst (0.5% *w*/*w* respect to polyol) mechanically stirred for 60 s were added with IPDI prepolymer (as reported, OH:NCO 1:1.1 molar ratio) and stirred for further 2 min; ^b^ Estimated error in the range of ±2–8; ^c^ Estimated error in the range of ±2.

**Table 2:** Diisocyanate Effect in Polyurethane preparation with Alcupol R3810/Borchi^®^ Kat 22 ^a^.

Run	Diisocyanate (g)	Product	*Tg* (°C) ^b^	WCA (°) ^c^	d (kg/dm^3^)
1	pure IPDI (17)	**PU_3810/K22/IPDI**	68	79	1.11
2	H12MDI (19.7)	**PU_3810/K22/H12MDI**	65	77	1.06
3	HMDI (12.6)	**PU_3810/K22/HMDI**	19	72	0.77

^a^ Formulation conditions: Alcupol R3810 (20 g), diisocyanate (1:1.1 OH/NCO), Borchi^®^ Kat 22 (2 g, 10% *w*/*w* respect to polyol), cure time = 12 h; ^b^ Estimated error in the range of ±2–8; ^c^ Estimated error in the range of ±2.

**Table 3:** Formulation of soy-based BIOPU_%C from PU_3810/K22/H12MDI ^a^.

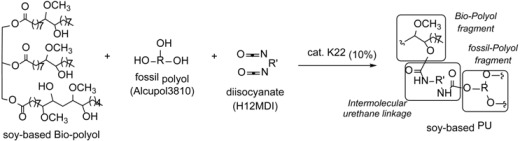								
Polyols Mixture g		Diisocyanate H12MDI (g)	Kat-22 (g) (10% *w*/*w*)	% of Bio-C ^b^	Bio-PU Products	*Tg* ^c^ (°C)	WCA ^d^ (°)	d (kg/dm^3^)
Alcupol R3810	Bio-Polyol							
20.2	4	19.8	2.42	11	**BIOPU_11**	73	90	1.06
9.6	4	10.2	1.36	19	**BIOPU_19**	62	75	1.05
9.2	6	10.4	1.52	25	**BIOPU_25**	49	76	1.06
8	8	11.3	1.68	31	**BIOPU_31**	48	82	1.08
Domes resin		IPDI prep	PhHg decanoate	0	**PU_MD ^e^**	59	77	1.01

^a^ Formulation according to procedure in experimental sections; ^b^ Determined according to Equation (1) in Section 3.4; ^c^ Estimated error in the range of ±2–8; ^d^ Estimated error in the range of ±2; ^e^ For preparation of **PU_MD** see experimental section.

**Table 4:** Petrochemical polyether polyols Eigenmann & Veronelli.

Commercial Name	n. OH (mgKOH/g)	Density (kg/dm^3^)	Viscosity (cP)
Alcupol C5710	570	1.05	700
Alcupol R3810	380	1.03	350
Alcupol R2510	250	1.02	260
Alcupol R1610	160	1.02	250
Alcupol C3531	35	1.05	800
Alcupol C2831	28	1.06	1100
